# P2X4 Receptors Mediate Ca^2+^ Release from Lysosomes in Response to Stimulation of P2X7 and H_1_ Histamine Receptors

**DOI:** 10.3390/ijms221910492

**Published:** 2021-09-28

**Authors:** Sin-Lih Tan, Muruj Barri, Peace Atakpa-Adaji, Colin W. Taylor, Ewan St. John Smith, Ruth D. Murrell-Lagnado

**Affiliations:** 1 Department of Pharmacology, University of Cambridge, Cambridge CB2 1PD, UK; sinlihtan21@gmail.com (S.-L.T.); pa376@cam.ac.uk (P.A.-A.); cwt1000@cam.ac.uk (C.W.T.); es336@cam.ac.uk (E.S.J.S.); 2 AstraZeneca, Aaron Klug Building, Granta Park, Cambridge CB21 6GH, UK; 3 School of Life Sciences, University of Sussex, Brighton BN1 9QG, UK; mb356@sussex.ac.uk

**Keywords:** P2X4, lysosomes, Ca^2+^

## Abstract

The P2X4 purinergic receptor is targeted to endolysosomes, where it mediates an inward current dependent on luminal ATP and pH. Activation of P2X4 receptors was previously shown to trigger lysosome fusion, but the regulation of P2X4 receptors and their role in lysosomal Ca^2+^ signaling are poorly understood. We show that lysosomal P2X4 receptors are activated downstream of plasma membrane P2X7 and H_1_ histamine receptor stimulation. When P2X4 receptors are expressed, the increase in near-lysosome cytosolic [Ca^2+^] is exaggerated, as detected with a low-affinity targeted Ca^2+^ sensor. P2X4-dependent changes in lysosome properties were triggered downstream of P2X7 receptor activation, including an enlargement of lysosomes indicative of homotypic fusion and a redistribution of lysosomes towards the periphery of the cell. Lysosomal P2X4 receptors, therefore, have a role in regulating lysosomal Ca^2+^ release and the regulation of lysosomal membrane trafficking.

## 1. Introduction

The P2X4 receptor is a member of the ATP-gated cation channel family. It is widely distributed in peripheral tissues and the central nervous system [1,2,3,4]. Genetic ablation of P2X4 receptors in mice has effects throughout the body, from elevated blood pressure and impaired long-term potentiation (LTP) to reduced cardiac contractility and pain behavior [5,6,7,8,9,10,11]. Upregulation of P2X4 receptors in macrophages and microglia enhances the release of prostaglandins and brain-derived neurotrophic factor, which contribute to the development of inflammatory and neuropathic pain [8,12,13,14,15,16]. Signaling downstream of P2X4 receptors is generally considered to be mediated by Na^+^ and Ca^2+^ influx through plasma membrane receptors. However, P2X4 has a unique distribution not shared by other members of the P2X family. In addition to its expression at the plasma membrane, it is predominantly localized to endolysosome membranes, with its ATP-binding sites located in the lumen [17,18,19]. ATP is accumulated within lysosomes by a voltage-sensing solute carrier (SLC17A9/VNUT) [20,21]. Given the high luminal concentrations of ATP, P2X4 receptors might be expected to regulate ion fluxes across lysosome membranes [20,22].

Lysosomes are an important Ca^2+^ store with an estimated luminal Ca^2+^ concentration of ~0.5 mM [23]. Release of Ca^2+^ from the lysosome is the trigger for lysosome fusion with other organelles, including endosomes, phagosomes, and autophagosomes, and this plays a critical role in the degradation of material from the endocytic and autophagic pathways. Lysosomes also fuse with the plasma membrane to release their contents extracellularly, and this is reportedly involved in processes as diverse as wound healing, tumor progression, cellular clearance, neurite outgrowth, and synaptic plasticity [24,25,26,27,28,29,30]. Several endolysosomal ion channels have been identified which are permeable to Ca^2+^ and Na^+^ [23,31,32]. Identifying a role for these channels in the fusion and fission of lysosomal compartments is confounded by the difficulties associated with the direct manipulation and recording of intracellular ion channel activity. There is, however, a precedent for P2X-like receptors controlling both the release of Ca^2+^ and fusion of acidic vacuoles. P2X-like receptors (*Dd*P2XA-E) expressed by *Dictyostelium* reside exclusively on the contractile vacuole and promote Ca^2+^ release and fusion of vacuoles with the plasma membrane as part of a cycle for regulating cell volume [33,34].

The high Ca^2+^ permeability of P2X4 receptors makes them candidates for mediating endolysosomal Ca^2+^ release, and accumulating evidence supports a role for them in regulating lysosomes. First, patch-clamp recordings from enlarged, vacuolar endolysosomes identified P2X4 receptor-mediated currents sensitive to both luminal ATP and pH, consistent with the known topology of the receptor [17]. Second, alkalinization of lysosomes using a weak base or inhibitor of the vacuolar-type H^+^ ATPase (V-ATPase) triggers P2X4 receptor-mediated endolysosome fusion via a calmodulin-dependent process [35]. Third, P2X4 receptors on specialized secretory lysosomes in type II alveolar cells mediate a Ca^2+^ signal after formation of a fusion pore between these organelles and the plasma membrane [36,37]. The Ca^2+^ signal then drives efficient secretion of luminal surfactant [38]. Furthermore, recent evidence in support of P2X4 regulating lysosome function was obtained in mouse models of two different pathologies: hepatectomy and experimental autoimmune encephalomyelitis (EAE) [39,40]. In the former, genetic ablation of P2X4 altered the distribution and secretion of lysosomes in hepatocytes [39], whilst, in the latter, potentiation of P2X4 using the positive allosteric modulator ivermectin triggered endolysosome fusion and enhanced the phagocytic capacity of microglia [40].

To identify physiological regulators of endolysosomal P2X4 receptors, we focused on two different signaling pathways: the P2X7 receptor and the H_1_ histamine receptor, the latter being an example of a G-protein-coupled receptor that stimulates Ca^2+^ release from the endoplasmic reticulum (ER) through IP_3_ receptors. The P2X7 receptor is a Ca^2+^-permeable channel in the plasma membrane, commonly co-expressed with P2X4, whose activation is reported to alkalinize lysosomes [41,42]. Whilst the high concentration of ATP in the lysosome lumen is important for activation of P2X4 receptors, the typically acidic pH within the lumen (pH ~4.5) inhibits P2X4 receptors [17,35,43]. A trigger for P2X4 receptor activation is, therefore, thought to be an increase in lysosomal pH. Ca^2+^ release via IP_3_ receptors provides the main source of Ca^2+^ taken up into lysosomes; therefore, stimulation of this pathway provides a way of testing the relationship between lysosomal Ca^2+^ uptake and P2X4 receptor activation. We show that stimulation of either P2X7 or H_1_ receptors can recruit lysosomal P2X4 receptor activity. A P2X4-dependent amplification of cytosolic Ca^2+^ signals occurred in the vicinity of lysosomes and this synergism between P2X4 and P2X7 was sufficient to trigger enlargement and redistribution of lysosomes, indicative of fusion. Ca^2+^-induced Ca^2+^ release (CICR) is a recurrent theme in Ca^2+^ signaling [44], but it is usually mediated by IP_3_ or ryanodine receptors. Our results suggest another CICR mechanism that links plasma membrane receptors to Ca^2+^ release from lysosomes through P2X4 receptors.

## 2. Results

### 2.1. P2X4 Receptors Are Expressed on Acidic Endolysosomal Compartments

**Figure 1:**
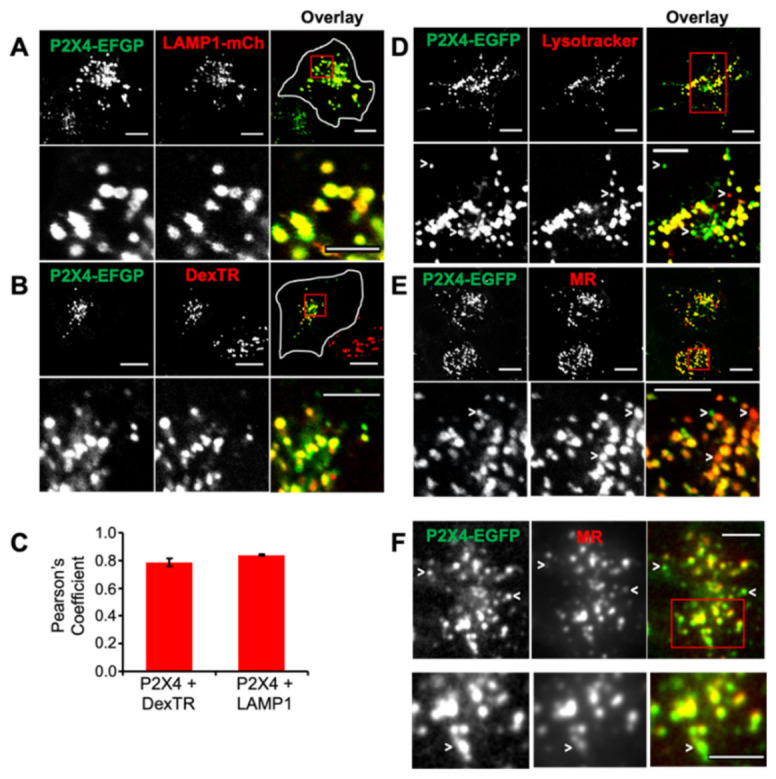
Colocalization of P2X4 receptors with endolysosomal markers. rP2X4-EGFP receptors were expressed in NRK cells, and live cells were imaged by confocal fluorescence microscopy 48 h post transfection after co-labeling lysosomes with a variety of different markers. These include (**A**) LAMP1-mCherry (LAMP1-mCh), which was co-transfected with rP2X4-EGFP, and (**B**) Texas Red-labeled Dextran (DexTR) incubated for 5 h and chased for 2 h at 37 °C. (**C**) Calculation of Pearson’s coefficient showed the high degree of colocalization between rP2X4 and LAMP1-mCh/DexTR. Results are the mean ± SEM from three independent experiments, total number of cells, *n* = 40. (**D**) Cells were incubated with Lysotracker (50 nM) for 10 min at 37 °C and, in (**E**,**F**), with a fluorescent substrate of cathepsin B, Magic Red^TM^ (MR) for 5 min. Cells in (**F**) were imaged using TIRF microscopy to show only those rP2X4- and MR-positive compartments located within ~100 nm of the plasma membrane. Arrowheads indicate examples of compartments that are labeled only by rP2X4 or MR. Scale bars are 10 µm (5 µm for enlargements).

Endolysosomes are heterogenous, and their pH varies from 4.5 to neutral [45]. Confocal images of live normal rat kidney (NRK) fibroblasts expressing P2X4-EGFP receptors showed colocalization between P2X4 and two lysosomal markers, LAMP1-mCherry (Figure 1A) and Texas Red Dextran (DexTR, Figure 1B); in both cases, Pearson’s coefficient values were ~0.8 (Figure 1C). Similar results were obtained in HeLa cells (Figure S1), and these results are also in agreement with our previous findings for both tagged and untagged P2X4 receptors and for native P2X4 receptors in immune cells [18]. To report the pH of endolysosomes, cells were incubated with either Lysotracker Red (LR, Figure 1D) or a membrane-permeable substrate of cathepsin B, Magic Red (MR, Figure 1E), which fluoresces after hydrolysis by cathepsin B and, therefore, also reports luminal acidity. Confocal images showed that most compartments containing P2X4 receptors were co-labeled with LR and MR. A minority of compartments containing P2X4 receptors did not appear to be acidic or contain active cathepsin B and vice versa (arrow heads in Figure 1D–F). We used total internal reflection fluorescence (TIRF) microscopy to image lysosomes immediately beneath the plasma membrane because these are reportedly less acidic than perinuclear lysosomes. Many of the near-plasma membrane compartments containing P2X4-EGFP receptors were MR-positive (Figure 1F, Pearson’s coefficient = 0.68 ± 0.48), but a considerable number were not. This is reflected in Mander’s correlation coefficient, which was lower for pixels that were positive for P2X4 receptors that also contained MR (0.47 ± 0.05) relative to its value for MR-positive pixels that also contained P2X4 (0.79 ± 0.03, *p* < 0.05, *n* = 3, 10 cells each). These results indicate that P2X4 receptors are expressed in heterogenous endolysosomal compartments.

### 2.2. Activation of Plasma Membrane P2X7 Receptors Increases Lysosome Size in a P2X4 Receptor-Dependent Manner

**Figure 2:**
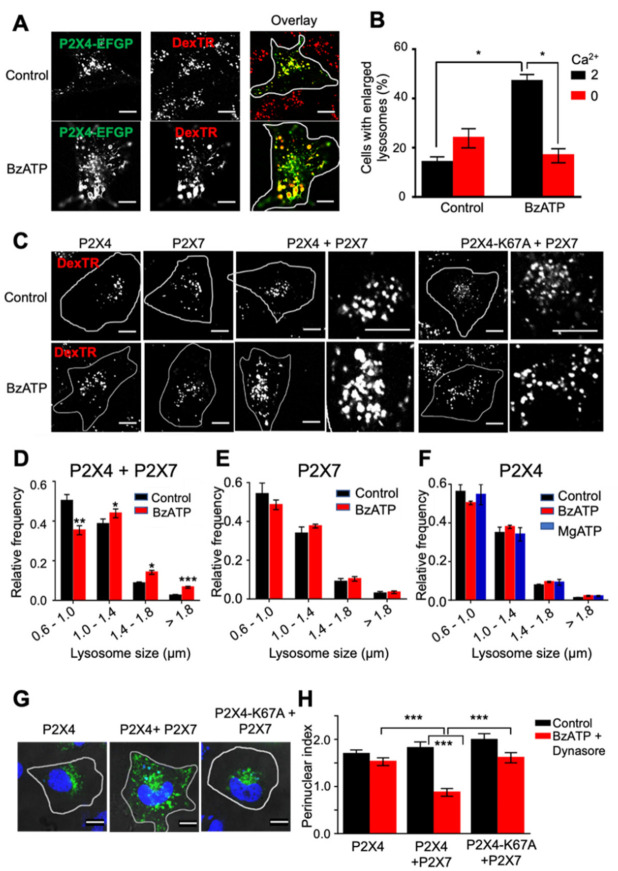
Activation of plasma membrane P2X7 receptors increases endolysosome size and trafficking in a P2X4 receptor-dependent manner. (**A**) In NRK cells expressing rP2X4-EGFP and rP2X7 receptors, endolysosomes were labeled with DexTR prior to treatment with 30 µM BzATP for 0.5 h at 37 °C. (**B**) A histogram of the percentage of cells with two or more enlarged DexTR positive lysosomes (≥1.8 µm) for control and BzATP-stimulated conditions. Stimulation with BzATP was carried out in normal DMEM (2 mM Ca^2+^) or zero Ca^2+^ DMEM. (**C**) Representative images of DexTR-labeled lysosomes in cells expressing the human isoform of P2X7 alone, in combination with rP2X4-EGFP, the rP2X4K67A-EGFP mutant, or rP2X4-EGFP alone. Control cells and those treated with 100 µM BzATP in DMEM for 0.5 h at 37 °C are shown. In cells expressing hP2X7 alone, EGFP was co-expressed to identify transfected cells. The frequency distribution of lysosome size for control and BzATP-stimulated cells is shown for cells co-expressing rP2X4 and hP2X7 receptors (**D**), expressing hP2X7 alone (**E**), and expressing rP2X4 alone (**F**). For cells expressing rP2X4 alone, a comparison was also made with cells treated with MgATP (100 µM) for 0.5 h at 37 °C. For statistical analysis, control versus agonist-stimulated was compared for each of the size groups. (**G**) To investigate lysosome trafficking, cells expressing either rP2X4 alone or co-expressing either wild-type rP2X4 or rP2X4-K67A with hP2X7 were treated with BzATP (100 µM, 0.5 h) and dynasore (80 µM, 0.5 h), fixed, and immunostained with an anti-P2X4 antibody and a FITC-labeled anti-rabbit secondary antibody. The nucleus was stained with DapI. (**H**) The distribution of rP2X4 was assessed by calculating the perinuclear index, which provides a measure of the proportion of receptors in the vicinity of the nucleus (≤5 µm) compared to those with a peripheral distribution (≥5 µm). All results are the mean ± SEM from three independent experiments. * *p* < 0.05, ** *p* < 0.01, and *** *p* < 0.001; one-way ANOVA followed by Tukey’s analysis. All scale bars, 10 µm.

Alkalinization of lysosomes with a weak base enhances P2X4 receptor-dependent formation of enlarged vacuolar lysosomes, indicative of homotypic lysosome fusion [35]. To test whether plasma membrane P2X7 receptors could trigger a similar response, rat P2X7 and P2X4 receptors were co-expressed in NRK cells, and endolysosomes were labeled with DexTR. Incubation with the P2X7 receptor-selective agonist, BzATP (30 µM, 30 min), increased the number of enlarged vacuolar lysosomes, an effect that was dependent upon extracellular Ca^2+^ (Figure 2A,B). Prolonged stimulation of rat P2X7 receptors can cause cellular toxicity; therefore, we also tested human P2X7 receptors which do not show the same degree of sensitization in the presence of agonist and, therefore, cause less cell toxicity [46,47,48]. In cells co-expressing P2X7 and P2X4 receptors, BzATP significantly shifted the frequency distribution of DexTR-labeled lysosome size toward larger lysosomes, whereas, in cells expressing just one of the receptors, there was no shift in the distribution (Figure 2C–F). As a further control, we tested two nonfunctional mutants of P2X4 co-expressed with P2X7. These two mutants, K67A and C353W, have a similar endolysosomal distribution to the wild-type receptor, but the shift in the distribution of lysosome size following BzATP treatment was not observed (Figure S2A,B). In similar experiments performed in HeLa cells co-expressing P2X4 and P2X7 receptors, the increase in the size of P2X4-positive organelles triggered by BzATP was inhibited by preincubation with the P2X7-selective antagonist, A-740003 (1 µM) (Figure S2C). Altogether these results show that stimulation of plasma membrane P2X7 receptors triggers a P2X4-dependent change in endolysosome membrane traffic.

Visual inspection of confocal images suggested that lysosome enlargement was accompanied by reduced clustering of lysosomes around the nucleus (Figure 2C). To further investigate the redistribution of endolysosomes, the subcellular location of P2X4-EGFP-labeled compartments was compared with and without preincubation with BzATP. These experiments included the dynamin inhibitor dynasore, which we previously showed inhibits dynamin-dependent endocytosis of P2X4 receptors [49,50]. BzATP significantly reduced the perinuclear localization of P2X4-EGFP-labeled compartments when P2X7 was co-expressed with P2X4-EGFP, but had no significant effect in cells expressing P2X4-EGFP alone (Figure 2G,H). In cells expressing P2X7 with the nonfunctional P2X4-K67A-EGFP receptor, there was no shift in labeled compartments in response to BzATP (Figure 2G,H). Since endocytosis of P2X4 receptors was inhibited, the reduced perinuclear localization reflects an increase in anterograde trafficking of P2X4-positive endolysosomes after stimulation of P2X7 receptors.

### 2.3. Stimulation of P2X7 Receptors Promotes Alkalinization of Lysosomes and Activation of Lysosomal P2X4 Receptors

**Figure 3:**
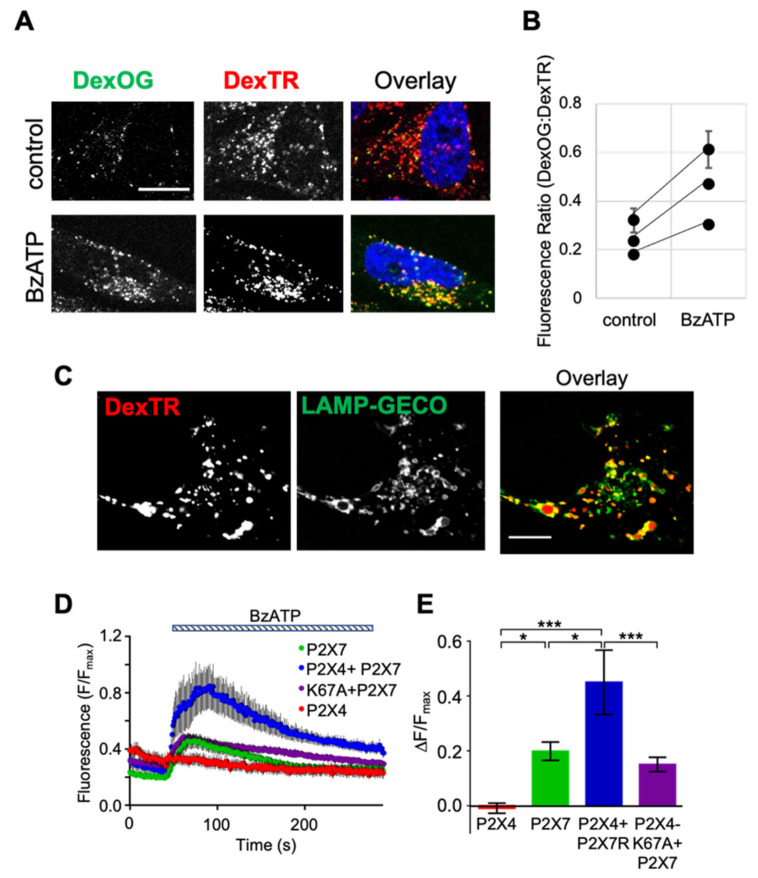
Stimulation of P2X7 receptors promotes endolysosome alkalinization and an enhanced P2X4 receptor-dependent Ca^2+^ signal. (**A**) Representative images of cells co-expressing rP2X4 and rP2X7 with endolysosomes labeled with the pH-sensitive Oregon Green 488 10 kD Dextran (DexOG) and pH-insensitive DexTR either with or without treatment with 30 µM BzATP for 0.5 h. (**B**) The fluorescence ratio of DexOG to DexTR per lysosome was increased in response to BzATP addition. The mean and SEM of this ratio for control and BzATP treated cells is shown for three independent experiments (557–2050 lysosomes per experiment). (**C**) Colocalization between LAMP1-GECO- and DexTR-positive compartments. (**D**) Changes in mean cellular LAMP1-GECO fluorescence during application of 30 µM BzATP in HBS is shown for cells expressing rP2X4 and rP2X7 receptors individually or together, or rP2X7 with the rP2X4K6A mutant, as indicated. The mean cellular fluorescence ± SEM was obtained from 50 cells per condition from a single experiment, and, for each cell, the response was normalized to the maximal response (F_max_) obtained by a final addition of ionomycin plus 5 mM Ca^2+^. (**E**) The mean peak amplitudes of the BzATP-evoked responses normalized to F_max_, from three separate experiments, are plotted for all of the receptor combinations. Results are the mean ± SEM. * *p* < 0.05, and *** *p* < 0.001; one-way ANOVA followed by Tukey’s analysis. All scale bars, 10 µm.

It has previously been reported that activation of P2X7 receptors triggers Ca^2+^-dependent alkalinization of lysosomal compartments [41]. Given the pH sensitivity of P2X4, we investigated if a similar shift in lysosomal pH occurred under the conditions of our experiments. In cells expressing P2X4 and P2X7, lysosomal compartments were loaded with a pH-sensitive Oregon Green-labeled Dextran (DexOG) together with the pH-insensitive DexTR. Incubation with BzATP increased the DexOG fluorescence such that the ratio of DexOG to DexTR fluorescence increased by 1.8 ± 0.16 fold (*n* = 3) compared to untreated cells (Figure 3A,B). Calibration of the ratio of DexOG to DexTR fluorescence as a function of lysosomal pH (Figure S3) suggests that this corresponds to an increase in lysosome pH of ~1.5 pH units. Similar to the findings of Guha et al. [41], P2X7 stimulated alkalinization was dependent upon extracellular [Ca^2+^] (Figure S3B).

To further investigate the role of P2X4 receptors downstream of P2X7 receptor activation, changes in cytosolic free [Ca^2+^] ([Ca^2+^]_c_) in the immediate vicinity of lysosomes were measured using a genetically encoded low-affinity sensor ($K_{d}^{\mathrm{Ca}}$ = 1.2 μM), GGECO, fused to the cytosolic tail of LAMP1 (LAMP1-GECO) [51]. The low affinity of this sensor ensures that local rather than global changes in [Ca^2+^]_c_ are reported. The targeting of LAMP1-GECO to lysosomes was shown by its colocalization with DexTR (Figure 3C). BzATP evoked changes in near-lysosome [Ca^2+^]_c_, and the peak amplitudes of these responses were significantly larger in cells co-expressing P2X4 and P2X7 receptors compared to cells expressing only P2X7 receptors or P2X7 with the P2X4-K67A receptor mutant (Figure 3D,E). As expected, there was no response to BzATP from LAMP-GECO in cells expressing P2X4 receptors without P2X7 receptors, consistent with BzATP being a poor agonist of P2X4 [4]. These results, showing that activation of P2X7 receptors evokes significantly larger near-lysosome cytosolic Ca^2+^ signals when functional P2X4 receptors are expressed, consistent with P2X4 receptors mediating Ca^2+^ release from lysosomes.

### 2.4. Stimulation of H_1_ Histamine Receptors Activates Lysosomal P2X4 Receptors

**Figure 4:**
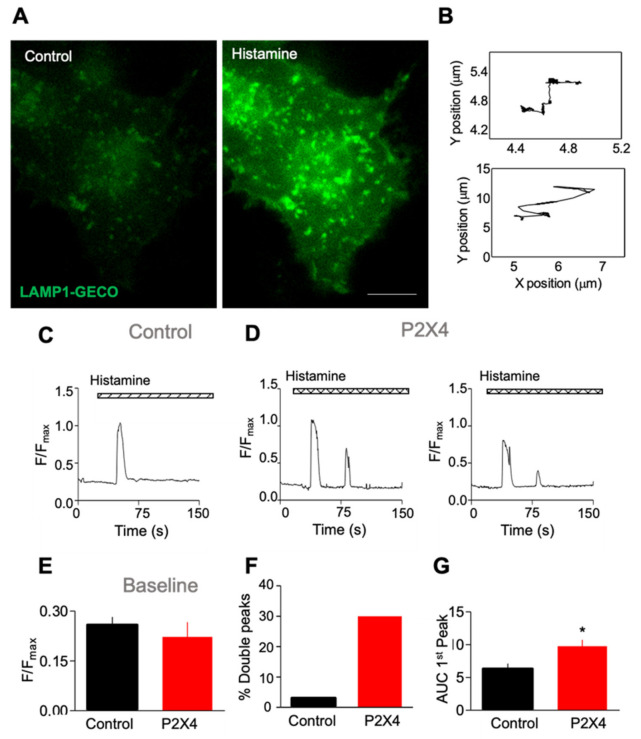
Expression of P2X4 alters histamine-evoked cytosolic Ca^2+^ signals measured at individual lysosomes. (**A**) Representative widefield images of HeLa cells co-expressing LAMP1-GECO with rP2X4 before and after stimulation with 50 µM histamine in Ca^2+^ free HBS. Scale bar = 10 µm. (**B**) Lysosomes were tracked during the experiment to elucidate [Ca^2+^]_c_ around single lysosomes, and examples of the trajectories of two different lysosomes that were tracked during a single experiment are shown. (**C**,**D**) Typical histamine-evoked responses recorded from single tracked lysosomes are shown for cells expressing LAMP1-GECO alone or co-expressing LAMP1-GECO with rP2X4. (**E**) There was no significant difference in the baseline fluorescence. (**F**) Percentage of tracks showing a double peak following stimulation with histamine (50 µM) was greater in cells co-expressing rP2X4 with LAMP1-GECO, as was the area under the curve for the first peak (**G**). The total number of tracks was 57 (LAMP1-GECO alone) and 63 (LAMP1-GECO with rP2X4) collected over 3–6 independent experiments; unpaired Student’s *t*-test, * *p* < 0.05.

Given the association of IP_3_ receptors with ER–lysosome contact sites [51], we wanted to determine if a receptor that stimulates IP_3_ production and the release of Ca^2+^ from the ER can exhibit functional crosstalk with lysosomal P2X4 receptors. Experiments were carried out in HeLa cells, which endogenously express histamine H_1_ receptors, and localized Ca^2+^ signals were again detected using LAMP1-GECO. To resolve the behavior of individual lysosomes, which move rapidly, LAMP1-GECO fluorescence was recorded from individual, tracked lysosomes (Figure 4A,B), and these responses came close to saturating the low-affinity indicator (Figure 4C,D). Baseline fluorescence was unaffected by expressing P2X4 receptors, but histamine stimulation evoked two temporally distinct peaks in ~30% of lysosomes, whereas a second peak was extremely rare in control cells (Figure 4C–F). In addition, the area under the curve (AUC) for the first peak was significantly larger in cells expressing P2X4 receptors compared to control cells (Figure 4G). These results suggest that P2X4 receptors release Ca^2+^ from lysosomes downstream of H_1_ receptor stimulation.

## 3. Discussion

Lysosomes are important intracellular Ca^2+^ stores, and lysosome-derived cytosolic Ca^2+^ signals regulate lysosome membrane trafficking and induction of autophagy genes [23]. P2X4 receptors regulate specialized secretory lysosomes in type II alveolar cells [37], but widespread expression of P2X4 receptors suggests additional roles. We provide the first demonstration that plasma membrane receptors can regulate endolysosomal P2X4 receptors. Using a low-affinity, targeted Ca^2+^ sensor to reveal peaks of [Ca^2+^]_c_ in the immediate vicinity of the lysosomes, we show that Ca^2+^ signals evoked by activation of P2X7 receptors are amplified by lysosomal P2X4 receptors. The resulting Ca^2+^ release from lysosomes causes anterograde trafficking and fusion of lysosomes. P2X4 also enhanced the near-lysosome Ca^2+^ response triggered by Ca^2+^ release from the ER through IP_3_ receptors, with individual lysosome tracking revealing an increase in the incidence of multiple discrete peaks. The regulation of lysosomal P2X4 by two unrelated receptors supports a widespread role for P2X4 in lysosomal signaling.

Lysosomes are heterogenous, with evidence that those near the plasma membrane are least acidic [52,53]. Our results show that P2X4 receptors populate heterogenous lysosomes, including those close to the plasma membrane and with increased pH. It seems likely that, during anterograde trafficking of lysosomes, the activity of P2X4 receptors will increase to control fusion of lysosomes with other compartments, including the plasma membrane. In NRK cells, lysosome exocytosis has been shown to be important for repair of the plasma membrane following wounding [30], and the delivery of the receptor and its ligand to the cell surface will also trigger further autocrine signaling. The mechanisms that link P2X7 and H_1_ receptor activation to the opening of endolysosomal P2X4 receptors remain unclear. Additionally, there might also be differences in the P2X4-dependent functional responses downstream of these receptors. A shift in pH has previously been shown to be sufficient to promote lysosomal P2X4 receptor activity because of the high levels of endolysosomal ATP [35]. We show here that stimulation of the P2X7 receptor produces an alkalinizing shift in lysosomal pH, and that this is dependent upon extracellular Ca^2+^. These findings are consistent with previously published results showing a P2X7-triggered increase in lysosome pH in retinal pigmented epithelial cells and microglia [41,42]. We do not, however, have evidence demonstrating that alkalinization of lysosome pH is necessary for activation of P2X4 downstream of P2X7 receptor stimulation, nor is it clear what the relationship is among a rise in [Ca^2+^]_c_, changes in lysosomal pH, and P2X4-mediated lysosome fusion. In NRK cells, direct elevation of [Ca^2+^]_c_ by the ionophore, ionomycin, was previously shown to trigger homotypic lysosome fusion [54], and we also showed that it could promote lysosome exocytosis and an increase in delivery of P2X4 and LAMP1 to the cell surface [18]. However, ionomycin mediates an increase in Ca^2+^ permeability of intracellular membranes, as well as the plasma membrane; therefore, it does not provide information about the coupling between receptor mediated increases in [Ca^2+^]_c_ and P2X4-dependent lysosome enlargement. In non-vertebrates, alkalinization of lysosomal pH has been directly linked to uptake of Ca^2+^ into lysosomes; however, a homolog of the vacuolar Ca^2+^–H^+^ exchange mechanism has not been identified in mammals. Furthermore, recent evidence suggests that, in mammalian cells, Ca^2+^ uptake into lysosomes is independent of the lysosomal pH gradient and instead dependent upon the voltage gradient across the lysosomal membrane [51,55]. Alkalinization is associated with the anterograde trafficking of lysosomes, a process that is controlled by lysosomal phosphoinositides [52]. It has also been suggested that, in macrophages, P2X7-dependent stimulation of phospholipase D is important for enhanced lysosome fusion with phagosomes [56]. Thus, in addition to increased [Ca^2+^]_c_, other pathways activated downstream of P2X7 receptor stimulation might be involved in P2X4-dependent regulation of lysosome membrane traffic.

We investigated functional coupling between the H_1_ receptor and lysosomal P2X4 because this is a G_q_-coupled receptor endogenously expressed in HeLa cells, but we think it likely that other G_q_-coupled receptors that stimulate Ca^2+^ release from the ER through IP_3_ receptors will behave in a similar manner. Because of the intimate association of lysosomes with the ER [51], the low-affinity LAMP1-GECO will detect Ca^2+^ release from the ER via IP_3_ receptors in addition to Ca^2+^ release from lysosomes. Therefore, the exact nature of the two distinct peaks of Ca^2+^ seen with increased frequency in the presence of P2X4, following H_1_ receptor activation, remains unclear. One possibility is that the first peak is primarily produced by Ca^2+^ release from the ER and the second peak is primarily the result of P2X4-mediated Ca^2+^ release from lysosomes, leading to a dramatic increase in frequency in cells expressing P2X4. An alternative explanation is that Ca^2+^-induced Ca^2+^ release from the ER contributes to the second peak, which is enhanced by the greater initial Ca^2+^ response contributed by a combination of Ca^2+^ release from the ER and P2X4-mediated Ca^2+^ release. The latter explanation would also explain the P2X4-dependent increase in the area under the curve of the first peak.

Our observations of P2X4-dependent changes in lysosome membrane traffic in NRK cells are consistent with our previous results obtained in macrophages and microglia [18]. In these immune cells, endogenous P2X4 colocalizes with LAMP1, and both proteins are upregulated at the cell surface following incubation of cells with a Ca^2+^ ionophore, a weak base, or an inhibitor of the lysosomal vacuolar-type H^+^ ATPase. Additionally, we showed that incubating peritoneal macrophages with 1 mM ATP to stimulate the endogenous P2X7 receptors increased secretion of the lysosome enzyme, beta hexosaminidase. However, it remains to be seen if knocking down expression of P2X4 in either macrophages or microglia disrupts lysosome membrane traffic.

The functional synergy between P2X4 and P2X7 receptors may account for several published observations. In breast cancer cells, P2X7 receptors promote secretion of lysosomal cysteine cathepsins and cancer cell invasiveness [57,58]. In immune cells, P2X7 receptor activation triggers the enhanced secretion of autophagolysosomal contents [42,59,60,61,62,63,64]. Another potential role for P2X4 and P2X7 receptors acting synergistically is in the clearance of pathogens following their phagocytosis. Stimulation of macrophage P2X7 receptors promotes lysosome fusion with phagosomes and aids killing of pathogenic bacteria which are normally protected because they inhibit phagosome maturation [56,65]. Genetic deletion of either P2X4 or P2X7 receptors substantially increased the sensitivity of mice to sepsis induced by pathogenic *Escherichia coli* and increased bacterial load in the blood [66].

We identified plasma membrane receptor-mediated pathways that recruit lysosomal P2X4 receptors, thereby triggering a lysosome-derived Ca^2+^ signal that contributes to regulation of lysosome trafficking and function. These lysosomal receptors are likely to be particularly important in cells with specialized secretory lysosomes and in cancer cells, which show enhanced lysosomal biogenesis and exocytosis.

## 4. Materials and Methods

### 4.1. Materials

Reagents were from Sigma-Aldrich (St. Louis, MO, USA) unless otherwise stated. Other reagents were obtained as follows: Dextran-Texas Red™ (DexTR; Mr 10,000, anionic, fixable), Dextran-Oregon Green 488 (DexOG; Mr 10,000, anionic, fixable), and LysoTracker DND 99 Red were from ThermoFisher Scientific (Paisley, UK). TransIT-LT transfection reagent was from Geneflow (Lichfield, UK), ionomycin from Apollo Scientific (Stockport, UK), polyethylenimine (PEI) from Polysciences (Warrington, PA, USA), PBS tablets from Gibco (Dublin, Ireland), Magic Red^TM^ kit from BioRad (Hercules, CA, USA), and nigericin (sodium salt) and monensin (sodium salt) from the Cayman Chemical Company (Ann Arbor, MI, USA). Anti-P2X4 antibody (1:250; cat number APR-002; Alomone Labs, Jerusalem, Israel) and Alexa 488 goat anti-rabbit (1:250; cat number A-11008; ThermoFisher, Paisley, UK) were used in immunocytochemistry experiments. The P2X7 receptor antagonist, A740003 was obtained from Tocris (cat number 3701). Sources of additional materials are provided within the relevant methods.

### 4.2. Plasmids

The construction and characterization of P2X4 receptors with enhanced green fluorescent protein fused to the C-terminus (P2X4-EGFP) has been described previously [49]. P2X4 and P2X7 receptors were also subcloned into the Clontech GFP-N1 vector using the NotI site to excise the coding sequence for GFP [46]. The K67A and C353W point mutations were generated in P2X4 and P2X4-EGFP using the QuikChange II site-directed mutagenesis kit (Stratagene, La Jolla, CA, USA). LAMP1-GECO was generated as previously described [51]. Briefly, cDNA encoding LAMP1-mCherry was amplified by PCR using oligonucleotide primers designed to introduce a *Hind*III site at the 5′ end. Similarly, cDNA encoding G_1.2_-GECO was amplified by PCR to introduce a *BamH*I site and an *EcoR*I site at the 5′ and 3′ ends of G_1.2_-GECO, consecutively. The amplified product of LAMP1-mCherry was excised using the *Hind*III site and *BamH*I, whereas the amplified G_1.2_-GECO was excised using the *BamH*I site and the *EcoR*I site. pcDNA3.1(+) was digested with *Hind*III and *EcoR*I overnight to create sticky ends suitable for ligation with the LAMP1 and G-GECO_1.2_ fragments. The digested LAMP1, G-GECO_1.2_, and pcDNA3.1(+) were ligated using T4 DNA ligase according to the manufacturer’s protocol (Thermo Fisher Scientific, Paisley, UK).

### 4.3. Cell Culture and Transfection

Normal rat kidney (NRK) cells and HeLa cells were maintained in Dulbecco’s modified Eagle’s medium (DMEM, Sigma-Aldrich, St. Louis, MO, USA, cat D6046) with 10% fetal bovine serum (FBS, Sigma-Aldrich, St. Louis, MO, USA) and 1% penicillin and streptomycin at 37 °C in humidified air with 5% CO_2_. For most imaging experiments, cells were plated onto glass coverslips coated with 1% (*w*/*v*) poly-l-lysine. For imaging individual lysosomes, HeLa cells were grown on 35 mm glass-bottom dishes (#P35G-1.0-14-C, MatTek Corporation, Ashland, MA, USA) coated with human fibronectin (10 µg/mL, Merck Millipore, Watford, UK). Transfections were carried out 24 h after plating using either PEI (Polysciences, Inc, Warrington, PA, USA) (1 µg DNA/2 µL of 1 mg/mL PEI) or TransIT-LT1 reagent (1 µg DNA/2.5 µL reagent, GeneFlow, Lichfield, UK). Cells were then incubated for 48 h at 37 °C prior to use.

### 4.4. Labeling Endolysosomes

For labeling lysosomes with DexTR, cells were incubated with 0.5 mg/mL DexTR for 5 h in DMEM at 37 °C, washed three times with PBS, and incubated for a further 2 h in DMEM without DexTR immediately prior to imaging. For labeling acidic compartments with Lysotracker, cells were incubated with 50 nM Lysotracker Red DND-99 at 37 °C in DMEM for 10 min and then washed three times immediately prior to imaging. For labeling with LAMP1-mCherry, cells were transfected with this construct at the same time as transfection with P2X4-EGFP and imaged 48 h later. Labeling of compartments for cathepsin B activity was carried out using the Magic Red^TM^ Cathepsin B kit according to the manufacturer’s instructions. Briefly, cells were incubated with the Magic Red (MR) reagent (1:26) for 5 min at 37 °C in DMEM and washed three times with PBS prior to imaging. In all cases, except where otherwise stated, cells were imaged using an oil-immersion 63× objective (numerical aperture, NA 1.40) using a Leica SP5 confocal microscope with excitation at 543 nm and emission bandwidth at 620–689 nm. For imaging P2X4-EGFP, the excitation wavelength was 488 nm, and the emission bandwidth was 495–532 nm. To obtain TIRF images (Figure 1F), coverslips were mounted on a TIRF microscope (IX51 inverted microscope (Olympus) with a 100× oil-immersion objective (NA 1.49) coupled to an electron-multiplying charge-coupled device camera (iXon; Andor Technology, Belfast, UK) and 488 nm argon ion and 561 nm diode lasers. MR-positive compartments were imaged at 561 nm (emission at 610–650 nm) and P2X4-EGFP-positive compartments were imaged at 488 nm (emission at 510–540 nm). For Pearson’s and Mander’s coefficient measurements, images were analyzed with Fiji/ImageJ using the JACoP plugin.

### 4.5. Analysis of P2X4 Receptor Trafficking

NRK cells were transfected with either P2X4, with or without P2X7, or P2X4-K67A plus P2X7. Then, 48 h post transfection, growth medium was changed to DMEM with or without BzATP (100 µM) and dynasore (80 µM), and cells were incubated for a further 30 min at 37 °C. After stimulation, cells were washed with ice-cold phosphate-buffered saline (PBS: 137 mM NaCl, 2.7 mM KCl, 10 mM Na_2_HPO_4_, 2 mM KH_2_PO_4_), fixed with paraformaldehyde (4%, 20 min) and permeabilized with saponin (0.5 mg/mL, 60 min, Sigma) in blocking solution (5% goat serum, 3% BSA in PBS). Cells were incubated with primary antibody in blocking solution (anti-P2X4, 1:250 dilution), washed twice with PBS, then further incubated in the dark with the secondary antibody (Alexa 488 goat anti-rabbit, 1:250), dried, mounted onto a glass microscope slide, and stored at 4 °C. Cells were imaged using an oil-immersion 63× objective (NA 1.40) using a Leica SP5 confocal microscope with excitation at 488 nm and emission bandwidth at 495–532 nm.

### 4.6. Analysis of Endolysosome Size and Distribution

Endolysosomes in NRK cells were labeled with endocytosed DexTR, as described above, and confocal images were analyzed using Fiji/ImageJ (Figure 2A–F, Figure S2B). After application of a threshold, particles which were considered as lysosomes had a circularity value of 0.7–1.0 A.U. The Feret diameter was used to report lysosome size, and, given the circularity of these organelles, this corresponds to an increase in lysosome volume. To measure the size of P2X4-positive compartments (Figure S2C), cells were transfected with P2X4-EGFP (Figure S2C). P2X4-EGFP-labeled compartments were analyzed in Igor Pro7 using SARFIA [67]. Regions of interest were selected using a threshold of 3× the standard deviation of all the pixel values in the Laplace operator. Only ROIs with an area >0.4 µm^2^ were included in the calculation of the geometric mean.

To assess the anterograde trafficking of P2X4-positive compartments toward the plasma membrane, the nucleus was labeled with DAPI (4′,6-diamidino-2-phenylindole), and the cell surface was visualized by bright-field imaging. The perinuclear region was defined as the region extending 5 µm from the perimeter of the nucleus, and the perinuclear index was calculated as follows: total cell fluorescence intensity (I_total_) = intensity of whole cell − nucleus intensity; perinuclear intensity (I_perinuclear_) = intensity ≤ 5 µm outward from nucleus − nucleus intensity; peripheral intensity (I_peripheral_) = intensity of whole cell − intensity that is ≤ 5 µm from nucleus; perinuclear index $=\frac{I_{\mathrm{perinuclear}}}{I_{\mathrm{peripheral}}}$.

### 4.7. Measuring Lysosome pH

Cells were incubated in DMEM containing both the pH-insensitive DexTR and the pH-sensitive DexOG for 5 h, followed by a 2 h chase in Dextran-free DMEM. Treatment with BzATP (30 µM) was carried out for 30 min at 37 °C, and cells were then washed with PBS and fixed using paraformaldehyde as described above, before mounting on glass coverslips. Coverslips were kept at 4 °C in the dark and imaged within 24 h using a Leica SP5 confocal microscope with settings for DexTR of excitation at 543 nm and emission bandwidth at 620–689 nm and for DexOG of 488 nm excitation and emission bandwidth at 498–547 nm. Images were analyzed using SARFIA in IgoPro7, and regions of interest were selected using a threshold of 3× the standard deviation of all the pixel values in the Laplace operator. ROIs were identified using the TR channel, and the background-corrected OG/TR fluorescence ratio for each organelle was calculated.

To generate a calibration curve, cells were loaded with DexTR and DexOG, and we used the method described in Bright et al. [53] to clamp lysosome pH over a range from pH 4 to pH 7. Solutions for pH clamping were as follows: for pH 4 and pH 5, 25 mM sodium acetate buffer; for pH 6, 25 mM MES buffer; for pH 7, 25 mM HEPES buffer, containing 5 mM NaCl, 1 mM CaCl_2_, 115 mM KCl, 1.2 MgSO_4_, 10 mM glucose, 10 µM nigericin, and 10 µM monensin. Live cells were incubated with these solutions for 5 min, and multiple images were taken. The background-corrected DexOG-to-DexTR fluorescence ratio was analyzed using SARFIA in IgoPro7 as described above.

### 4.8. Measuring Cytosolic Ca^2+^ Signals near Lysosomes

LAMP1-GECO was transfected into NRK and HeLa cells, and confocal imaging experiments were performed 48 h post transfection in HEPES-buffered saline (HBS) (140 mM NaCl, 5 mM KCl, 1 mM CaCl_2_, 1 mM MgCl_2_, 10 mM D-Glucose, 10 mM HEPES, pH 7.3). Cells were imaged using the Leica SP5 confocal microscope, with an oil-immersion 63× objective (excitation at 488 nm and emission bandwidth at 495–532 nm) and a time interval between frames of 0.65 s. F_max_ was obtained by addition of ionomycin (5 µM) in HBS containing 5 mM Ca^2+^. For those experiments where the Ca^2+^ response at individual lysosomes was measured, cells were imaged using used an inverted Olympus IX83 microscope, with a 100× objective (NA 1.49), excitation at 488 nm, and emission peak/bandwidth at 525/50 nm. Wide-field images were acquired at 50 frames/min, and F/F_max_ was obtained by the addition of ionomycin (10 µM) concurrently with 2 mM Ca^2+^. All fluorescence images were corrected for background by subtraction of fluorescence from a region outside the cell. Image capture and processing was done using MetaMorph Microscopy Automation and Image Analysis Software (Molecular Devices). Following background correction, the Ca^2+^ response around single lysosomes was assessed using single-particle tracking with the MetaMorph Track Objects plugin [68], using a Template-match algorithm to connect tracks between successive frames. All tracks were expressed as F (fluorescence at each timepoint) divided by the F_max_ (fluorescence following treatment with ionomycin).

### 4.9. Data Analysis

Most results are presented as the mean ± SEM from at least three independent experiments, as indicated in the figure legends. Statistical analysis was assessed using GraphPad PRISM with either an unpaired Student’s *t*-test or one-way ANOVA followed by either Tukey’s or Games–Howell post hoc test, as indicated in the figure legends.

## Supplementary Materials

The following are available online at https://www.mdpi.com/article/10.3390/ijms221910492/s1.
