# Supplementary material for: P2X4 Receptors Mediate Ca2+ Release from Lysosomes in Response to Stimulation of P2X7 and H1 Histamine Receptors
Source: Int J Mol Sci. 2021 Sep 28;22(19):10492. doi: 10.3390/ijms221910492 (PMC8508626; doi:10.3390/ijms221910492)
Supplement: Supplementary file 1 [file ijms-22-10492-s001.zip › ijms-1357833-supplementary.pdf]

International Journal of
Molecular Sciences

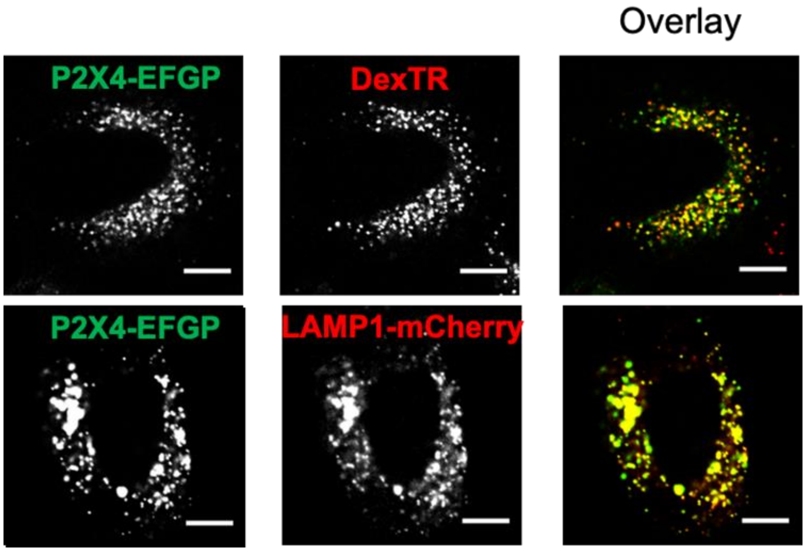

Figure S1. Endolysosomal targeting of P2X4 in HeLa cells. Confocal images of HeLa cells expressing rP2X4-EGFP receptors shows a high degree of colocalization with both LAMP1-mCherry and DexTR. Scale bars represent 10 μm.

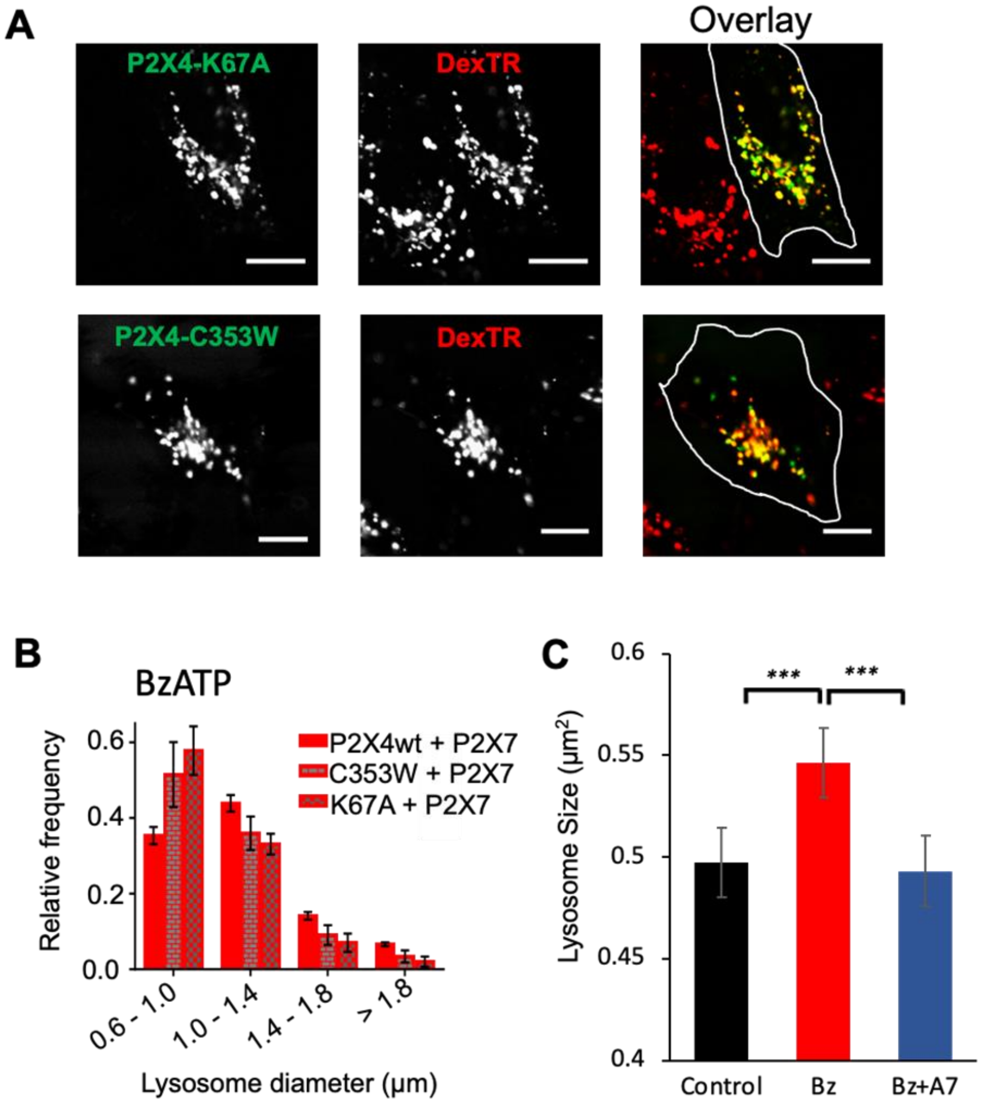

Figure S2. The synergistic action of P2X7 and P2X4 receptors promotes an increase in endolysosome size. (**A**) Two nonfunctional mutants of rP2X4, K67A and C353W, have a similar endolysosomal distribution to the wild-type rP2X4 receptor. (**B**) Comparison of the frequency distribution of DexTR-labeled lysosome size for cells expressing hP2X7 with either wild-type rP2X4 or the non-functional K67A and C353W mutants, following incubation with 100 µM BzATP for 30 min at 37 °C. NRK cells were used 48 h post transfection with the indicated receptors. Only DexTR-positive compartments >0.6 µm diameter were included in this analysis. (**C**) The P2X7 receptor antagonist A740003 inhibited the effects of BzATP on the size of P2X4-labeled compartments. HeLa cells expressing rP2X7 and rP2X4 receptors were incubated with either 30µM BzATP alone or following a 15 min preincubation with 1 µM A740003. All results are the mean ± SEM from three independent experiments. Statistical analysis was performed using one-way Welch ANOVA with Games–Howell post hoc test. Data are presented as the geometric means ± SEM with *** $p < 0.0001$ (*n* control = 1729; *n* BzATP = 2097; *n* BzATP+A7 = 1768).

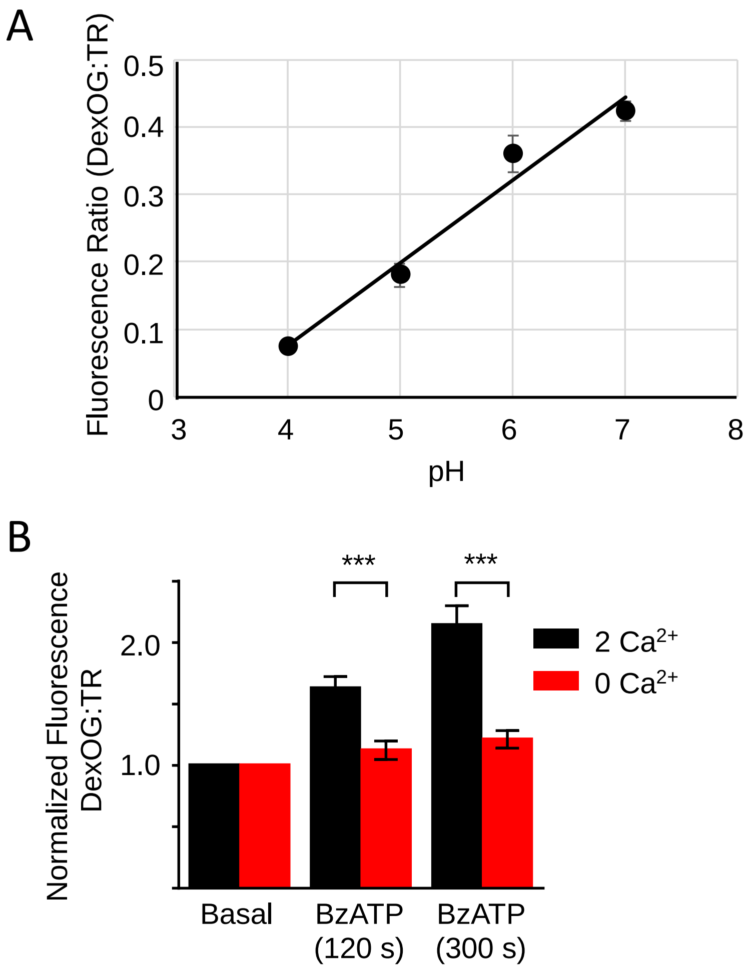

Figure S3. (**A**) Calibration curve for ratio of Oregon Green Dextran (OG) to Texas Red Dextran (TR) fluorescence as a function of luminal pH. In live cells preloaded with DexOG and DexTR, lysosomal pH was clamped at values between pH 4 and pH 7 using a range of different buffers in combination with nigericin (10 μM) and monensin (μM). (**B**) Cells co-expressing P2X4 and P2X7 were incubated with DexOG and DexTR and imaged at 20 °C in HBS or nominally Ca^{2+}-free HBS. The fluorescence ratio Dex OG:TR increased following 100 μM BzATP addition in a Ca^{2+}-dependent manner, as measured at the timepoints indicated.
